# A Novel Neurotrophic Drug for Cognitive Enhancement and Alzheimer's Disease

**DOI:** 10.1371/journal.pone.0027865

**Published:** 2011-12-14

**Authors:** Qi Chen, Marguerite Prior, Richard Dargusch, Amanda Roberts, Roland Riek, Cédric Eichmann, Chandramouli Chiruta, Tatsuhiro Akaishi, Kazuho Abe, Pamela Maher, David Schubert

**Affiliations:** 1 Cellular Neurobiology Laboratory, The Salk Institute for Biological Studies, La Jolla, California, United States of America; 2 Structural Biology Laboratory, The Salk Institute for Biological Studies, La Jolla, California, United States of America; 3 Laboratory of Pharmacology, Research Institute of Pharmaceutical Sciences, Musashino University, Nishitokyo-shi, Tokyo, Japan; 4 Molecular Neurosciences, The Scripps Research Institute, La Jolla, California, United States of America; 5 Laboratorium f. Physikalische Chemie, ETH Zurich, Zurich, Switzerland; Thomas Jefferson University, United States of America

## Abstract

Currently, the major drug discovery paradigm for neurodegenerative diseases is based upon high affinity ligands for single disease-specific targets. For Alzheimer's disease (AD), the focus is the amyloid beta peptide (Aß) that mediates familial Alzheimer's disease pathology. However, given that age is the greatest risk factor for AD, we explored an alternative drug discovery scheme that is based upon efficacy in multiple cell culture models of age-associated pathologies rather than exclusively amyloid metabolism. Using this approach, we identified an exceptionally potent, orally active, neurotrophic molecule that facilitates memory in normal rodents, and prevents the loss of synaptic proteins and cognitive decline in a transgenic AD mouse model.

## Introduction

At present, there are few drugs that improve the memory deficits associated with normal aging and none that prevent cognitive decline in chronic neurodegenerative conditions such as Alzheimer's disease (AD). Except for the rare cases of familial AD, the cause of AD is not known, but the disease is highly correlated with aging, a process involving a wide variety of physiological changes. Therefore, it is likely that the cells in the aging brain are compromised not from a single cause but from the convergence of multiple insults. However, the currently most widely used approach to drug discovery is to identify a single molecular target and then make a drug that alters this target [1]. Unfortunately, drugs for AD that were developed through this approach have all failed in clinical trials, perhaps because one target is not sufficient or because the targets are also critical for normal brain function so that their inactivation results in toxicity. Therefore, a different approach to drug discovery for AD is needed [2], [3].

Since age is the prime risk factor for AD as well as many other neurodegenerative conditions, we developed a drug screening procedure that is based upon old age-associated pathologies without requiring pre-selected molecular targets. A series of cell culture assays were brought together that mimic multiple pathways of CNS nerve cell damage and we required that a drug candidate show efficacy in all of these assays before it could be moved forward into AD animals. The assays include the loss of trophic support, oxidative stress, the reduction of energy metabolism (*in vitro* ischemia and glucose starvation) and amyloid toxicity. As potential lead drug candidates to screen in these assays, we chose a representative from the original pharmacopeia, plant-based polyphenolics. Natural products have a wide range of molecular targets because they resemble biosynthetic intermediates to a greater extent than synthetic drugs and are therefore able to compete with substrates for multiple enzymes [4].

Curcumin is a curry spice with multiple biological activities that is also effective in transgenic AD mouse models [5], [6]. To improve the potency and pharmacokinetic properties of curcumin, we synthesized a series of hybrid molecules between curcumin and cyclohexyl-bisphenol A (CBA), a compound that has neurotrophic activity which curcumin lacks [7]. The best compound in our initial library was CNB-001, a molecule that has improved stability over curcumin and that is neuroprotective in multiple neurotoxicity assays in which curcumin is inactive [8]. We then generated a large number of derivatives of CNB-001 and selected the best compound on the basis of activity in our multiple toxicity assays. The result was a much more potent molecule called J147. It was then asked if J147 is effective in two paradigms of age-associated pathology, AD and memory. It is shown here that this broadly neuroprotective compound has the ability to enhance memory in normal animals as well as to prevent memory deficits in AD transgenic mice. The neurotrophic and memory-enhancing activities of J147 are associated with an increase in brain derived neurotrophic factor (BDNF) levels and the expression of BDNF responsive proteins, the enhancement of LTP, synaptic protein preservation, the reduction of markers for oxidative stress and inflammation, the reduction of amyloid plaques, and lower levels of soluble Aβ_1–42_ and Aβ_1–40_. These pleiotrophic effects of a single molecule suggest that J147 has potential for the treatment of AD.

## Results

**Figure 1:**
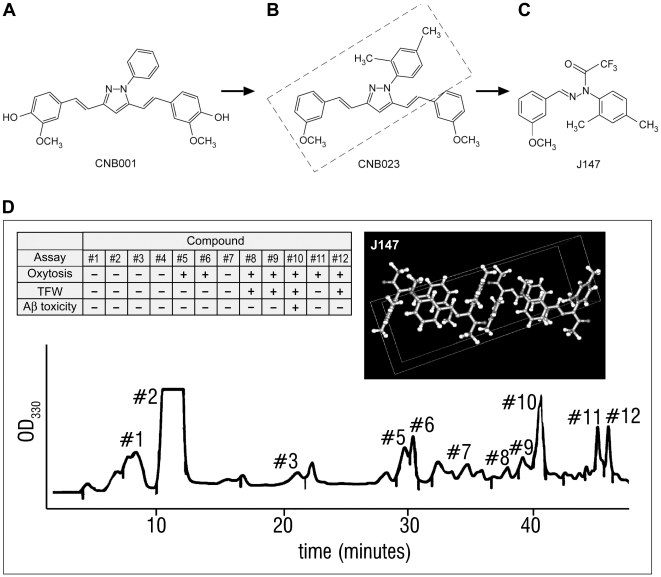
Selection of J147. CNB-001 (A) is a broadly neuroprotective and neurotrophic derivative of curcumin [8]. CNB-023 (B) is a derivative of CNB-001 lacking hydroxyl groups but with similar activity that was identified by SAR analysis of CNB-001. The boxed area shows the hypothesized biologically active fragment of CNB-023. A large collection of molecules around this chemical space was generated by the reaction of m-anisaldehyde with 2.4 dimethylphenyl hydrazine (see Materials and Methods) and J147 (C) was selected from the reaction products on the basis of its activity in trophic factor withdrawal (TFW), oxidative stress (oxytosis) and Aβ toxicity assays (see Figure 2). (D) Final HPLC run of reaction mixture that generated J147. The structure of the most active product (fraction 10) was determined by NMR. J147 was then synthesized, and its crystal structure (insert) determined, confirming its biological activity and structural identity. The following are de-risking and target identification screens, with no significant reproducible hits. J147 was used at 10 micromolar unless indicated. (1) LeadProfiling+P450 screen. Over 60 CNS receptors and transporters (work done by MDS Pharma). (2) hERG (work done by MDS and Absorption Systems). (3) Acute toxicity in rats. Negative at 2 grams/kilogram (work done by Absorption systems). (4) CeeTox “Safe” up to 90 micromolar plasma concentration (work done by CEETOX, INC). (5) 352 protein kinases (done by Ambit). (6) MDRI-MDCK brain penetration classification “High” (work done by Absorption Systems). (7) Enzyme Assays. Cox 1; Cox 2; Lox 5,12,15; Sirt 1,2,3; LT4 hydrolase; LTC4 synthase; cathepsin B; matrix metalloprotease 1; phosphodiesterases 10A1, 11A1, 1A, 2A, 3A, 4A1A, 4B1, 5A, 6, 7A, 7B, 8A1, 9A2; proteasome (done by MDS). (8) Enzyme assays. PPARα, γ; deubiquitinases, BAP1, UCH-L1, L3, USP5; acetylcholine esterase; MAOA; MAOB; phosphatases (19 of them), proteases and caspases (8) (done by Caliper).

**Figure 2:**
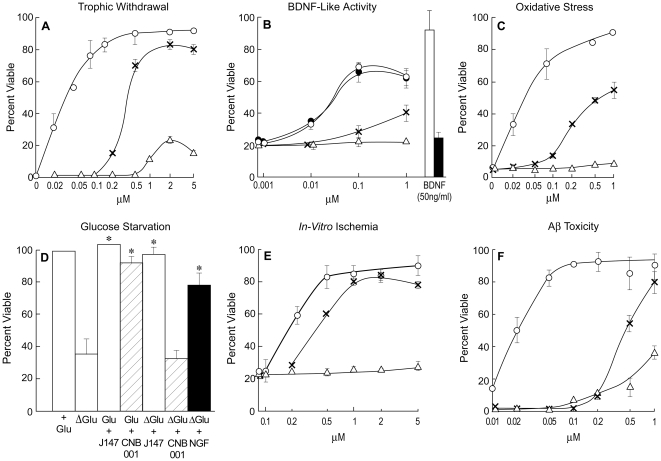
Biological Activities of J147. J147 is active with EC_50 s_ between 10 and 200 nM in six different assays for neurotrophic activity and neurotoxicity. o-o, J147; x-x, CNB-001; Δ-Δ, curcumin. (A) Trophic Factor Withdrawal. Primary cortical neurons were prepared from 18-day-old rat embryos and cultured at low cell density with or without the three compounds. Cell viability was assayed 2 days later. (B) BDNF-like Activity. HT22 cells expressing the TrkB (BDNF) (open circles, J147) receptor or no TrkB (black circles, J147) were placed in serum-free medium in the presence of 50 ng/ml BDNF or the indicated amounts of compounds. Cell viability was determined 2 days later. Curcumin had no activity in this assay up to one micromolar. BDNF was used at 50 ng/ml and active only in cells expressing TrkB (open bar), not in its absence (black bar). (C) Oxidative Stress. E18 rat cortical neurons were treated with 5 mM glutamate and different concentrations of compounds one day after plating when no ionotropic glutamate receptors are expressed. Cell viability was measured 24 hr later. (D) Glucose Starvation. PC12 cells were starved for glucose plus or minus 20 nM J147, 0.2 µM CNB-001 or 10 µM curcumin and cell viability determined 48 hr later. J147 and NGF increase cell viability in the absence of glucose, *P<0.001 vs. control. CNB-001 and curcumin are inactive at 0.2 and 10 µM respectively (curcumin not shown). (E) Chemical ischemia. HT22 cells were treated with 20 µM iodoacetic acid for 2 hr alone or in the presence of varying concentrations of J147, CNB-001 or curcumin. Percent survival was measured after 24 hr. (F) Amyloid toxicity. Primary hippocampal cells were exposed to 5 µM Aβ_1–42_ in the presence of increasing amounts of compounds and cell viability determined 48 hr later. All data shown are mean ± SEM, n = 3 or 4. The curcumin and CNB-001 data which were included for comparison with J147 have been presented, in part, previously [8], [84].

Because many of the pathological consequences of AD are enhanced features of the stresses that occur with old age [9], we devised a drug discovery scheme that uses multiple assays to test efficacy against these distinct stresses. We started with a multi-target natural product, curcumin [3], and required that synthetic derivatives have both improved medicinal chemical properties and lower EC_50_s in all of our assays of age-related stresses, not just one. Figure 1 summarizes this approach. Briefly, a pyrazole derivative of curcumin called CNB-001 (Figure 1A) is neuroprotective in multiple cell culture assays designed to reflect the various cellular toxicities associated with AD [8]. Figure 2 shows the assays that were used to select the best compounds and presents a comparison between curcumin and CNB-001. Because aromatic hydroxyl groups may be covalently modified in vivo by sulfation and carbohydrate addition thereby limiting brain penetration [10], derivatives of CNB-001 were synthesized that did not contain hydroxyl groups but still maintained its biological activities. A limited structure-activity relationship (SAR) analysis of CNB-001 showed that the hydroxyl groups are not necessary for activity and that activity was maintained following the addition of two methyl groups to the nitrogen-associated aromatic ring of CNB-001 (Fig. 1B, CNB-023. Table 1). To improve the pharmacological properties of CNB-023 and explore the possibility that two aromatic rings connected by a nitrogen containing bridge are sufficient for activity, a large number of molecules were synthesized in a single reaction, followed by the selection of the most active compound in the mix and the determination of its structure. To generate diversity, we carried out a reaction between 2,4 dimethylphenyl hydrazine and m-anisaldehyde under conditions which generate a wide variety of reaction products. The reaction products were then separated initially by thin layer chromatography (TLC) followed by reverse phase chromatography. Biological activity was followed by three primary rat cortical neuron culture assays that are detailed below; an assay for trophic factor activity [11]; [8], a glutamate-induced oxidative stress assay called oxytosis [12], and amyloid toxicity toward hippocampal neurons [13]. When the most active TLC fraction was separated on a HPLC column, only one peak was effective in all three assays (Fig. 1D). The structure of this compound was determined by NMR spectroscopy and confirmed by synthesis and crystal structure (Figs. 1C and D). It was designated J147 (Fig. 1C). J147 has the medicinal chemical properties of a good CNS drug candidate with respect to size (351 MW), cLogP (4.5), total polar surface area (41.9) and pharmacokinetics (not shown) [14]. Also included in the legend of Fig. 1 is an abbreviated list of assays used to identify both potential drug targets and screens to identify potential toxicities. To date there is no evidence for potential side effects, nor has a molecular target been identified. As detailed below, J147 also has a low EC_50_ in multiple neurotoxicity assays.

**Table 1:** Structure activity relationship of a few CNB-001 derivatives.

Compound	R_1_	R_2_	R_3_	Aβ - EC_50_	HT22 - EC_50_
CNB-001	OH	OMe	H	460 nM	1.8 µM
CNB-020	H	H	H	>10 µM	>10 µM
CNB-022	H	OMe	H	2 µM	5 µM
CNB-026	OMe	OMe	H	>10 µM	>10 µM
SK37H	H	OMe	4-Et	500 nM	1 µM
CNB-077	H	OMe	4-Me	400 nM	1 µM
	H	OMe	2,4-di-Me	400 nM	560 nM

The structure of CNB-001 is shown in Figure 1A. A series of CNB-001 derivatives were synthesized with the goal of removing the hydroxyl group and maintaining and/or improving biological activity in the glutamate based oxytosis assay and extracellular amyloid toxicity. Et (ethyl), Me (Methyl).

### J147 is Broadly Neuroprotective

Since the original lead compound, CNB-001, was selected on the basis of its broad neuroprotective activity [8] and J147 on the basis of a subset of these activities, it was first asked if J147 maintained all of the activities of CNB-001. Figure 2 shows that J147 is biologically active in six assays that represent distinct neurotoxicity pathways related to aging and neurodegenerative disease. In one assay of trophic factor withdrawal (TFW), primary embryonic cortical cells are plated at low density in serum-free medium (Fig. 2A). At low density, the cells die within two days but can be rescued by combinations of neurotrophic growth factors, but not by one alone [[11] and unpublished]. In contrast, cell death is prevented by J147 alone with an EC_50_ of 25 nM. In a second assay, J147 is able to rescue a clone of the hippocampal nerve cell line HT22 expressing the BDNF receptor, transmembrane receptor kinase B (TrkB), from serum starvation under conditions where the cells can be protected by BDNF (Fig. 2B) [15]. J147 is equally neuroprotective using cells lacking TrkB, showing that J147 does not require the BDNF receptor for activity. Figure 2C shows that J147 also rescues primary cortical neurons from oxytosis, an oxidative stress-induced programmed cell death pathway caused by glutathione (GSH) depletion [12]. A reduction in GSH is a common denominator of old age and essentially all chronic CNS diseases [16]. J147 is also neuroprotective against glucose starvation (Fig. 2D) and the loss of energy metabolism in an in vitro ischemia model (Fig. 2E) [17], [18]. Finally, J147 is able to block extracellular amyloid toxicity using rat hippocampal neurons with an EC_50_ of about 200 nM (Fig. 2F). J147 does not, however, bind to and directly inhibit Aβ_1–42_ aggregation using the thioflavin S assay (not shown), as do its precursors curcumin and CNB-001 [8], [6]. Curcumin is only marginally active in two neuroprotection assays [8], the trophic factor withdrawal and Aβ toxicity assays, at the indicated concentrations (Fig. 2 A and F), while CNB-001 is significantly less effective than J147 in all assays. These data show that our drug discovery scheme is effective at identifying a highly potent neuroprotective molecule. J147 has neurotrophic and BDNF-like activities that are independent of the BDNF receptor, TrkB (Fig. 2B). BDNF is a molecule that is also involved in promoting memory, is reduced in the brain with age, and in AD as well as in other neurological disorders [19], [20], [21], and it has long been considered a drug target for AD [22].

### J147 Enhances Long-Term Potentiation and Memory

**Figure 3:**
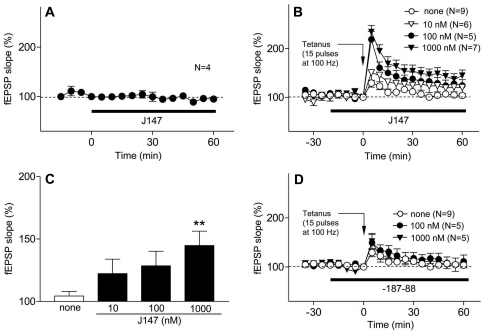
J147 Facilitates the Induction of LTP in Schaffer Collateral CA1 Pyramidal Cell Synapses in Rat Hippocampal Slices. (A) Effect of J147 (1 µM) on basal synaptic transmission. Hippocampal slices were exposed to J147 during the time indicated by the black bar. The fEPSP slope is expressed as the percentage of the value immediately before the addition of J147. J147 does not affect basal synaptic transmission. (B) J147 facilitates the induction of LTP after a weak tetanic stimulation (15 pulses at 100 Hz) which alone does not induce LTP in control slices. The effect of J147 is concentration-dependent. Time course of changes in the fEPSP slope. The hippocampal slices were untreated (o, n = 9) or exposed to J147 (▾, 0.01 µM, n = 6; ▴, 0.1 µM, n = 5; •, 1 µM, n = 7) for the time indicated by the black bar and weak tetanic stimulation was applied at time 0. The fEPSP slope is expressed as the percentage of the value immediately before the application of weak tetanic stimulation. (C) Concentration-dependency. To compare the data among the groups, the averages of the fEPSP slopes 30–60 min after tetanic stimulation were calculated as an index of LTP magnitude. J147 demonstrated a concentration dependent effect with 1000 nM having the greatest effect on the fEPSP slope (n = 7 slices per rat: one-way ANOVA F(3,23) = 4.4, **P = 0.01). (D) Negative Control. −187–88, the alkene form of J147 in which nitrogens are replaced by carbons (o, n = 9; ▴, 0.1 µM, n = 5; •, 1 µM, n = 5) showed no effect. All data shown are means ± SEM.

Given the association between memory, BDNF and AD, we first asked whether or not J147 could affect long-term potentiation (LTP) in hippocampal slices. LTP is considered a good model of how memory is formed at the cellular level. Although J147 had no direct effect on basal synaptic transmission in the CA1 area of rat hippocampal slices (Fig. 3A), it induced LTP in slices exposed to a weak titanic stimulation (15 pulses at 100 Hz), which by itself failed to induce LTP (Fig. 3B). The LTP facilitating effect of J147 was concentration-dependent in the range of 0.01–1 µM (Fig. 3C). An inactive analog of J147 (−187–88) in which the two nitrogens were replaced by carbons had no activity in this assay (Fig. 3D).

**Figure 4:**
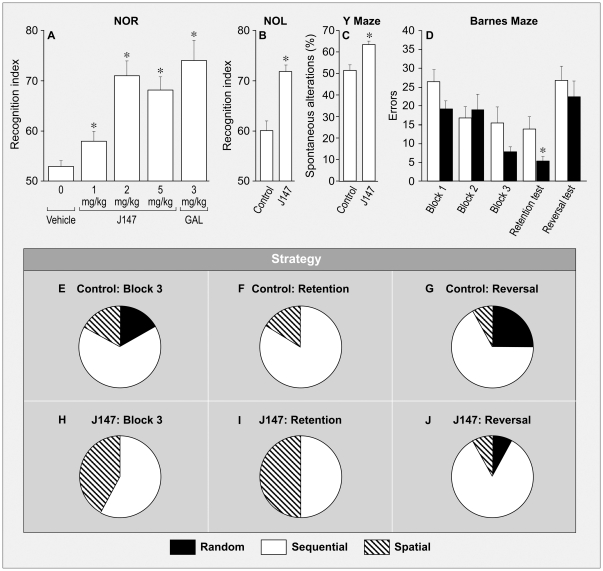
Behavioral Assays. (A) The novel object recognition test (NOR) was employed for compound J147. Doses of 1, 2 and 5 mg/kg were administered by oral gavage to 7 wk old Sprague-Dawley rats 1 hr prior to training. Treatment with J147 increased the recognition index with all three concentrations. Galantamine, injected IP at 3 mg/kg, served as a positive control, one-way ANOVA F (3, 40) = 12.9. *P<0.05 n = 12 per group (Done by Behavioral Pharma, La Jolla, CA). In panels B-J normal male C57BL/6J mice were fed J147 at 200 ppm for 2 wks and the Barnes maze assay was then done, which required 8 weeks to complete. This was followed by the novel object location (NOL) and Y-maze assays. (B) Novel object location (NOL) assay; the assay was performed similarly to (A) except that one object was used and it was moved to a new location instead of being replaced by another. J147 treated mice had significantly greater recognition indexes than control mice in this assay (n = 12 per group: one-way ANOVA F(1,22) = 2.5, *P<0.05). (C) Y-maze. The tendency of rodents to sequentially explore 3 arms of the Y-maze is a measure of short-term memory. While the groups did not differ in total arm entries, mice fed J147 exhibited a higher percentage sequential entries into all 3 arms (spontaneous alterations). F(1,22) = 3.3 p = 0.0032. (D) The error rate in the acquisition phase of the Barnes maze, 9 consecutive days, pooled data from 3 days per block. A lower error rate indicates better learning. The difference between control (white bar) and J147-fed mice (black bar) in Blocks 1 and 3 are almost significant (n = 12 per group: one-way ANOVA F(1,22) = 2 and F(1,22) = 1.8, respectively, *P*>0.05 for both). Six weeks after the final acquisition phase, mice were re-assayed and J147 significantly improved memory (retention test) (n = 12 per group: one-way ANOVA F(1,22) = 2.6, *P<0.01) but had no effect when the escape tunnel was relocated (reversal test). (E–J) The strategy used by the mice to find the escape tunnel was determined from videotapes and mice fed J147 tended to use a spatial strategy rather than sequential or random ones (z = 2.33, *P* = 0.019 for Block 3; z = 1.73, *P* = 0.80 for retention). All data shown are means ± SEM.

It has recently become possible to identify early presymptomatic stages of AD in which there is minimal cognitive impairment, the best time to initiate treatment [23]. Current measures of outcome in AD clinical trials require a significant improvement in cognition for FDA approval, but none of the recently developed drugs showed such an effect in clinical trials and may require very long treatment durations to achieve significance. Therefore, we asked if J147 is active in promoting learning and memory following a short-term treatment using multiple assays in normal animals. These assays include the novel object recognition (NOR) test in rats, and the novel object location (NOL) test, Barnes and Y mazes in mice; they all measure various aspects of spatial learning and memory. The innate exploratory nature of rodents can be used to assess memory using the NOR and NOL tests [24], for both require habituation where the rodent becomes familiar with objects in certain locations. The habituation period is followed by the testing period where the animal is exposed to a novel object in a known location or a known object in a novel location. In the NOR test, during the habituation period rats were presented with two identical objects, which they explored for a fixed time period. Memory is assessed one day later when two objects are presented, one of which was presented previously during the training and is thus familiar; while the other object is new to them. It is hypothesized that memory for the familiar object results in more time exploring the novel object. J147 was administered orally to the rats before the start of the habituation period to assess its effects on memory, specifically consolidation of the familiar object. Figure 4A shows that oral administration of J147 only one hour prior to the habituation period enhanced memory of the familiar object in the rat NOR test. There was a statistically significant increase in the recognition index following all 3 doses of J147. A derivative of J147 that was inactive in the LTP assay, −187–88 (Fig. 3D), was also inactive in the NOR test (not shown). The positive control for this assay was the acetylcholinesterase inhibitor, galantamine, injected IP at 3 mg/kg. J147 is not, however, an acetylcholinesterase inhibitor or a phosphodiesterase inhibitor (PDI) as assayed against acetylcholinesterase and 12 PDIs at 10 µM, (MDS Pharma Services, not shown). The inhibition of both enzymes promotes memory and both are drug targets for memory enhancement [25], [26]. Therefore, the mode of action of J147 on memory may be distinct from the traditional memory enhancing drugs.

To explore the ability of chronic J147 treatment to stimulate various aspects of memory in young mice, they were fed J147 at 200 ppm (10–20 mg/kg/day) in their chow. After 2 wks on J147 chow, they were sequentially assayed in the Barnes maze, Y-maze and NOL. The NOL test relates to spatial short-term recognition memory where the exploration of a novel object over repeated trials and its subsequent change in location assays the ability of mice to build up spatial representations of their environment. J147 treated mice demonstrated a greater difference between the final trial with the old location and the trial with the new location (*P* = 0.0006 vs. *P* = 0.017) yielding a significant difference in the recognition index (F(1,22) = 2.5, *P*<0.05; Fig. 4B). This result demonstrates that J147 treatment resulted in an improvement in spatial memory in these mice.

To assay the ability of J147 to alter another aspect of memory, we used the Y maze. The spontaneous tendency to alternate free choices in entering the 3 arms of the Y maze is a measure of short-term memory [27], [28]. The groups did not differ in overall numbers of arm entries, suggesting that activity levels were not altered by J147 treatment. However, J147 treated mice made a higher percentage of spontaneous alternations (defined as consecutive entries into the 3 different arms) in this test F(1,22) = 3.3, *P* = 0.0032) (Fig. 4C). These data indicate that J147 improves short-term memory.

The Barnes maze assay is also a spatial learning and memory test [29]. It is less stressful and physically taxing than the Morris water maze and suitable for studies in all ages and abilities of mice. In addition, the strategies used by the animals to perform the task are readily revealed. Mice were trained to find an escape tunnel among 20 choices in a circular platform for 3 blocks of 3 days each for the acquisition phase. The retention test for long-term memory was done 6 weeks later with the escape tunnel in the same position. The escape tunnel was then moved to a new location and the behavior of the mouse recorded (the reversal test). Although there was no statistically significant differences between groups (F(1,22) = 2.9, *P*>0.05), there were trends toward decreased errors in the J147 group in block 1 (F(1,22) = 2.0, *P* = 0.057) and block 3 (F(1,22) = 1.8, *P* = 0.086) trials in the acquisition phase (Fig. 4D). Importantly, a greater proportion of J147-treated mice used a spatial strategy to locate the escape chamber in block 3 (z = 2.33, *P* = 0.019) (Fig. 4E and H). This difference appeared to hold during the retention (memory) test (Fig. 4F, I), where the J147 treated mice made significantly fewer errors than control mice (F(1,22) = 2.6, *P* = 0.01) (Fig. 4D). There was no group difference in errors made when the escape chamber was moved in the reversal test (Fig. 4D). However, a greater number of J147 mice utilized a sequential strategy during this test (Fig. 4G, J), suggesting that they adopted a working memory strategy in the face of change. J147-treated mice used more optimal strategies in the Barnes maze, resulting in superior spatial memory as evidenced in the retention test. Therefore, J147 meets the initial criterion of a drug for AD in that it has the ability to enhance several different aspects of memory with short-term treatment in multiple animal models.

### J147 Prevents Memory Deficits in an Alzheimer's Disease Animal Model

**Figure 5:**
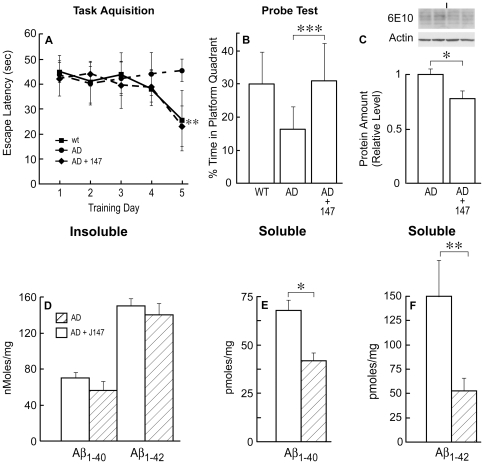
J147 Improves Learning and Short-Term Spatial Memory. Line 85 mice were fed J147 at 200 ppm between 3 and 10 months of age and then assayed for learning and memory in the Morris water maze. (A) Task acquisition phase. Data are expressed as standard deviation ± SEM and analyzed by ANOVA of repeated measures. F(2,14) = 8.3. **P<0.01 AD vs AD+J147; *P*<0.05 AD vs. control. (B) Probe test. One-way ANOVA F(2,54) = 16.6. **P<0.001 AD vs AD+J147; *P*<0.05 AD vs. control. These data show that both wild type and line 85 mice are slow learners, but that J147 corrects the deficit in both learning (day 5) and their spatial memory (probe test). (C) Aβ oligomers between 30 and 50 kD in soluble fraction from AD (left 2 lanes) and AD+J147 (right 2 lanes) animals. J147 treatment decreased these oligomers. Two-tailed *P* value, *P<0.05. Aβ_1–40_ and Aβ_1–42_ levels were measured by ELISA in control AD animals (open bars) and AD animals fed J147 (hatched bars) in the insoluble (100,000×g pellet) (D) and RIPA soluble fractions (E and F). J147 treatment decreased both soluble Aβ_1–40_ and Aβ_1–42_ levels. Two-tailed *P* values *P<0.05; **P<0.01; *** *P*<0.001. All data shown are means ± SEM. n = 6 to 9 mice per group.

Since J147 is orally active in memory assays in normal animals, we asked if it could prevent the behavioral deficits in a transgenic mouse model of AD. The line 85 APPswe/PS1/ΔE9 transgenic AD mouse (huAPP/PS1) was used because there is an extensive literature on their pathology and behavior [30], [31], [32], [33]. There is good pathology (plaques and loss of synaptic markers) at 9 months along with clear deficits in the Morris water maze (MWM) assay. The transgenic mice were fed J147 in their chow at 200 ppm starting at 4 mo of age. This dose was chosen based upon the potency of J147 in the memory assays (Fig. 4). Since we already had extensive behavioral data on J147 in wild type rodents, control animals plus J147 were not included in these experiments. Mice were assayed for memory deficits in the MWM and for pathological and biochemical changes after 5 additional months on J147 or control diet. In the acquisition task portion of the MWM, an assay for learning [34], the transgenic mice showed deficits that were corrected by treatment with J147 (Fig. 5A). Since these were older animals, even the non-transgenic littermate control mice were relatively slow learners [35]; [36]. However, by day 5 the mice with J147 diet had learned the task, while the untreated AD mice did not. In the probe trial, an assay for spatial memory, there was also significant improvement, with the J147 diet restoring memory of the platform location to control levels (Fig. 5B).

### J147 Reduces Soluble Aβ Levels in the Hippocampus

**Figure 6:**
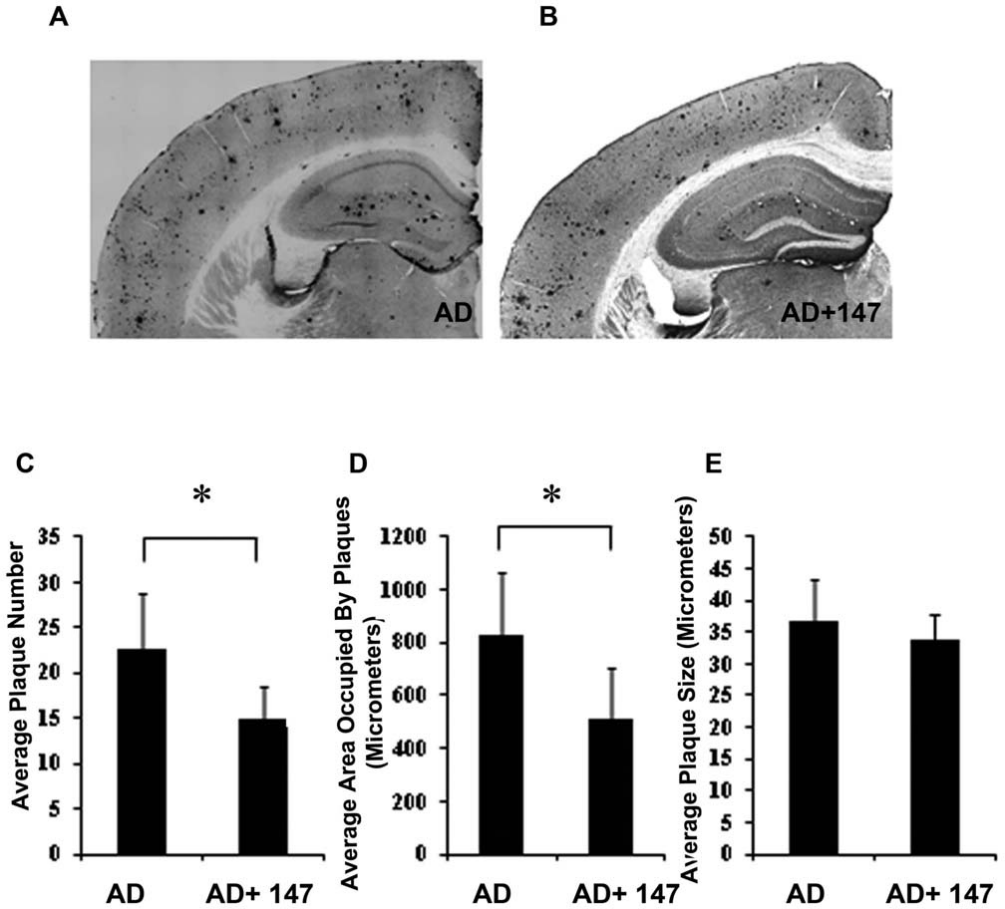
Immunohistochemical analysis of condensed Aβ deposits. Immunohistochemical analysis was done using brain coronal sections of 13-month old AD mice on control diet (A) or J147 diet (B) using antibody 6E10. In comparison to AD control mice (A), condensed Aβ deposits were significantly reduced in the hippocampus region of AD mice on J147 diet (B). 30 micron thick sections of similar regions from each mouse, (n = 6) were examined and plaque counts in the hippocampus were quantified. All images were quantified using Image J Software. (C) The average of plaque counts for each mouse group is expressed as a number of plaques ± the SD (n = 6 per group: unpaired t-test, *P<0.05). (D) Reduced Aβ plaque load in AD mice on J147 diet. The Aβ plaque load was determined by measuring the area occupied by Aβ-immunoreactive condensed deposits, (n = 6 per group: unpaired t-test, *P<0.05). (E) The plaque size did not change between AD mice on control and J147 diets (n = 6 per group: unpaired t-test, *P>0.05).

Since some compounds that reduce memory loss in AD mice reduce Aβ plaque burden, we next examined plaque size and density as well as Aβ levels in the RIPA insoluble (100,000×g pellet) and soluble (RIPA supernatant) fractions of the hippocampi of J147 treated and control huAPP/PS1 mice. Neither Aβ_1–42_ nor Aβ_1–40_ levels as measured by ELISA, were altered in the RIPA insoluble fraction in animals fed J147 relative to untreated AD transgenic animals (Fig. 5D). Although there was no difference in plaque size between control and J147 treated animals, the average area occupied by plaques and the total number of plaques in each group was significantly different (Fig. 6). Therefore, while treatment with J147 enhanced the cognitive ability of the huAPP/PS1 mice, it had no significant effect on insoluble Aβ levels but a small but significant reduction of plaque load. However, it is now thought that soluble Aβ polymers are major contributors to the toxicity associated with the peptide [37]. Figure 5E and F show that the J147 diet reduces the amount of RIPA soluble Aβ_1–40_ and Aβ_1–42_ in the hippocampus and Figure 5C demonstrates a reduction in Aβ polymers, detected by the 6E10 antibody, in the 40–60,000 molecular weight range in the transgenic mice fed J147 relative to control animals on a drug-free diet. It follows that J147 has a small but significant effect on Aβ metabolism by reducing Aβ_1–42_ in the soluble fraction of the hippocampus.

### J147 Reduces Oxidative Stress and the Inflammatory Response

**Figure 7:**
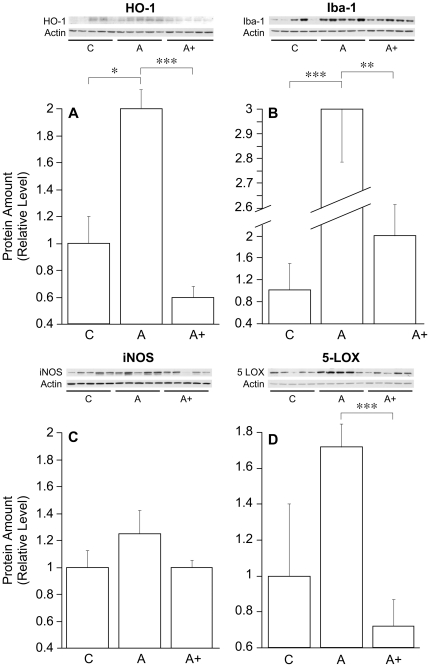
J147 Reduces Pro-oxidant Enzymes in the Brains of huAPP/PS1 mice. Cell lysates of hippocampal tissue from huAPP/PS1 and control mice were analyzed by western blotting and the images quantified. C = control; A = AD transgenic; A+ = AD transgenic fed J147. (**A**) Heme oxygenase-1 is increased in AD transgenic mice compared to control and these levels are decreased in mice fed J147. (B) Iba-1 levels are decreased in AD transgenic mice fed J147. (C) iNOS levels are not quite significantly decreased in AD transgenic mice fed J147. *P* = 0.053. (D) 5-LOX is significantly decreased in AD transgenic mice fed J147. *P<0.05, **P<0.01, and ***P<0.001. All data shown are means ± SEM, n = 5 per group.

As outlined in the Introduction, the biochemical profile of the brain changes with age and many pathological aspects of the aged brain are enhanced in AD. Specifically, there is an increase in pro-oxidants and markers of oxidative stress as well as inflammation, and these have been associated with a decline in cognitive skills, both in AD and normal aging [38] [39]. At the same time, cells in the AD brain are trying to survive the pathological insults associated with the disease by increasing their neuroprotective mechanisms. It is the relative balance of the two processes that determines the fate of the individual cell. A successful AD drug should alter this balance in favor of survival, returning expression levels of the molecules involved to control values. To determine if J147 is able to alter any markers for oxidative stress in huAPP/PS1 transgenic mice, Western blots of hippocampal tissue were carried out to determine the expression levels of several stress-related proteins. Figure 7A shows that there is a large increase in heme oxygenase 1 (HO-1) in huAPP/PS1 mice that was decreased to control levels by J147. HO-1 is frequently thought of as an antioxidant enzyme [40], but it is elevated in AD brain and can act as a pro-oxidant under some conditions [41]. Microglia mediate a significant part of the inflammatory immune response in the CNS and are activated and increased in number in AD [39]. Biochemical markers for microglia include Iba-1 and inducible nitric oxide synthase (iNOS). Figure 7B shows that there is a significant reduction in the amount of Iba-1 in the hippocampus of J147-treated huAPP/PS1 mice relative to control huAPP/PS1 mice while the reduction of iNOS is not quite statistically significant due to the large mouse to mouse variability with this marker (Fig. 7C). Finally, 5-lipoxygenase (5-LOX) is responsible for the synthesis of leukotrienes that are potent mediators of inflammation and chemotaxis of immune cells [42], and are overexpressed in AD [43]. 5-LOX is highly upregulated in the huAPP/PS1 mice and brought down to control levels by J147 (Fig. 7D). Together these data suggest that J147 is able to modulate the level of oxidative stress and the inflammatory response in huAPP/PS1 mice.

### J147 Reduces Heat-Shock Proteins and Increases Synaptic Protein Expression

**Figure 8:**
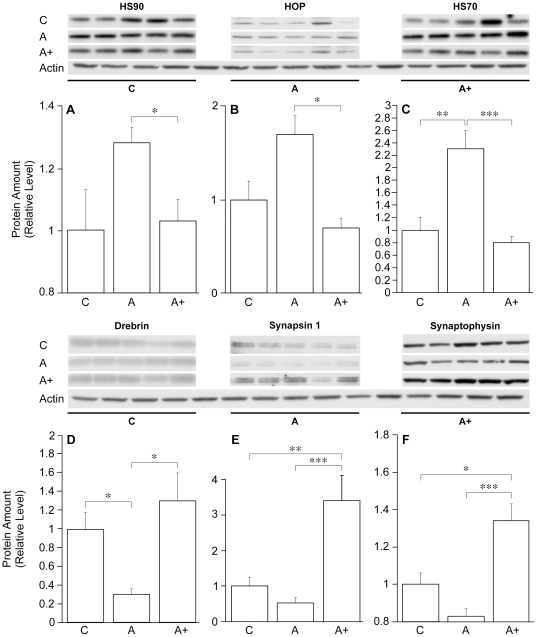
J147 Reduces Heat Shock Protein Stress Response and Increases Markers for Synaptic Function. Cell lysates from hippocampal tissue of line 85 mice fed J147 for 7 months were analyzed by Western blotting. C = control; A = AD transgenic; A+ = AD transgenic fed J147. (A) HSP90 levels are decreased in AD transgenic mice fed J147. (B) HOP levels are decreased in AD transgenic mice fed J147. (C) HSP70 levels are increased in AD transgenic mice and these levels are decreased with treatment of J147. (D) Drebrin levels are decreased in AD transgenic mice and J147 treatment restores levels to above control levels. (E) Synapsin-1 levels are decreased in AD transgenic mice and are restored significantly beyond control by J147. (F) Synaptophysin levels are decreased in AD transgenic mice and levels are restored beyond control levels with J147. *P<0.05, **P<0.01), and ***P<0.001. All data shown are means ± SEM, n = 5 per group.

Oxidative stress damages proteins and degrades their ability to fold properly. To compensate, cells increase the synthesis of proteins that promote proper folding, including members of the large heat shock family. In AD, there is a large increase in the expression of the heat shock family of chaperones and the co-chaperone HOP [44]. Figure 8A–C shows that the levels of HSP70, 90 and the co-chaperone HOP are increased in huAPP/PS1 mice and decreased to control levels by J147.

To obtain some insight into the structural basis of the protection from cognitive loss afforded by J147, the expression of three major synaptic proteins was examined. Drebrin, synapsin 1 and synaptophysin were chosen because their loss has been correlated with both human AD and behavioral deficits in transgenic mouse lines [45]; [46]; [47]. Figure 8D–F shows that all three proteins are reduced in huAPP/PS1 mice and their levels brought to control levels or higher by J147.

### BDNF is Up-Regulated by J147

**Figure 9:**
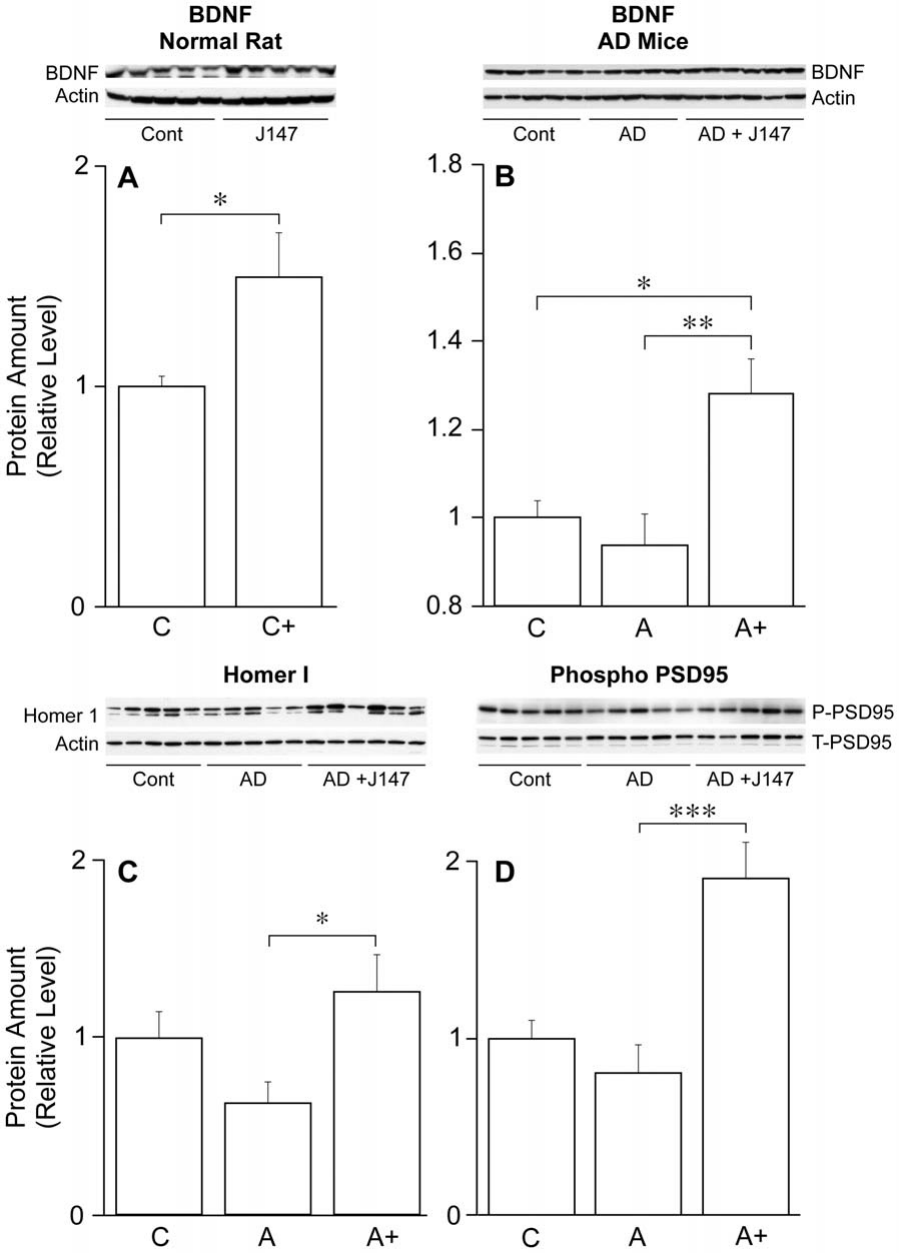
J147 Increases the Levels of BDNF and BDNF Responsive Protein and Protein Phosphorylation. (A) BDNF expression in the hippocampus of normal adult rats following two weeks treatment of J147 in food at 200 ppm. J147 treatment increased BDNF levels in the normal rat. Two-tailed t test. *P<0.05. (B) BDNF expression in hippocampus of huAPP/PS1 transgenic mice fed J147 for 7 months before sacrifice at 10 months. BDNF levels are decreased in AD transgenic mice and treatment with J147 restores levels to beyond control levels. (C) Homer-1 expression in huAPP/PS1 mice. J147 treatment increases Homer-1 levels. (D) Phosphorylation of PSD95 at Tyr 236, 240. J147 treatment increases phosphorylation of PSD95 in AD transgenic mice. Data are presented as ratio of phospho PSD95 to total PSD95. *P<0.05, **P<0.01, and ***P<0.001. All data shown are means ± SEM. n = 5–6 per group. C = control; C+ = control + J147; A = AD transgenic; A+ = AD transgenic fed J147.

J147 was synthesized and selected for its ability to act as a neurotrophic factor in part using assays where it can replace the function of BDNF. It was therefore asked if J147 is able to increase the expression of BDNF and/or activate one or more of its downstream signaling pathways. J147 increases the levels of BDNF in the hippocampus of both normal rats (Fig. 9A) as well as in huAPP/PS1 transgenic mice (Fig. 9B). BDNF directly regulates the expression of several genes involved in synaptic spine formation as well as the phosphorylation of proteins involved in learning and memory [48]. Homer 1 is an actin binding protein that is induced by BDNF [49]. Figure 9C shows that J147 restores the expression of this protein relative to untreated huAPP/PS1 mice. The phosphorylation of the scaffold protein PSD95 is mediated by BDNF [50]. Figure 9D shows that J147 greatly increases the phosphorylation of PSD95 at Tyr 236 and 240 in huAPP/PS1 mice relative to both control and untreated AD mice. J147 does not, however, require the expression of the BDNF receptor TrkB for its neuroprotective activity (Fig. 2B) and the levels of TrkB in the AD mice are not altered by J147 (not shown). Together these results show that J147 both increases BDNF levels and modulates the expression and/or phosphorylation of downstream targets of BDNF.

## Discussion

Familial AD (FAD) is caused by mutations in a small subset of genes that control the generation of Aβ [51]. Because of these findings, it is widely believed that Aβ is the causative factor in AD and the vast majority of the drug development effort for AD is focused upon Aβ or components of the Aβ biosynthetic pathway. While Aβ is clearly relevant, over 95% of all AD cases are not FAD and age is by far the greatest risk factor for the disease. It is therefore likely that the various pathological components of old age such as oxidative stress, the loss of trophic factors, reduced energy metabolism, and inflammation all lower the threshold for Aβ toxicity. It follows that conditions that reduce these stressors are likely to delay the onset of AD and/or reduce its consequences [3]. These age-associated parameters have not, however, been employed as a group for targeting pathways in AD drug discovery.

To explore the possibility of using these parameters for the discovery and development of AD drugs, we initially synthesized a library of hybrid molecules between the multi-target plant polyphenolic curcumin and a neurotrophic molecule, cyclohexyl-bisphenol A. We then selected a compound called CNB-001 that was neuroprotective in six toxicity assays [8]. Selection was followed by a limited structure-activity (SAR) analysis of CNB-001, and a chemical reaction that was able to generate extensive diversity around the structural components of CNB-001 that the SAR suggested were required for activity. From the products of this reaction, we identified one compound, J147, that was active in the original assays but that had EC_50_s up to 100 times lower than CNB-001 in the various selection assays. J147 also has greatly improved medicinal chemical properties for a CNS drug.

Since two of the six selection assays are directly for neurotrophic activity, and BDNF is a neurotrophic factor that is reduced in AD brain and is required for normal memory, we asked if J147 could modulate hippocampal LTP, memory, and prevent behavioral deficits in a huAPP/PS1 AD mouse model. Figure 3 shows that J147 is able to facilitate the induction of LTP in hippocampal slices. LTP requires a large number of signaling cascades, including those activated by BDNF [48]. Like J147, exogenous BDNF promotes, but does not cause LTP [52]. Conversely, LTP is attenuated in multiple strains of BDNF knockout mice [48]. LTP is associated with the increased expression of BDNF and of the BDNF responsive gene, Homer 1. BDNF also increases the phosphorylation of PSD95. J147 mimics BDNF by both maintaining Homer 1 expression and increasing the phosphorylation of PSD95 in AD transgenic mice (Fig. 9 C and D). Because J147 has both long- and short-term biological effects, as well as in multiple cell culture aging/disease models, it is likely that efficacy is the result of pleiotrophic mechanisms. For example, BDNF-like neuroprotective activity does not require the expression of the BDNF receptor (Fig. 2B), but it is able to induce BDNF expression *in vivo*. In addition, the *in vivo* metabolites of J147 have the same biological activities as the parent, which may contribute to its long-term efficacy (in preparation).

The normal aging process is often associated with a reduction in learning and memory, so it would be very useful for an AD drug to also enhance non-pathological memory loss. Several different types of memory tests that assess aspects of memory in humans can be performed in rodents [53]; [54]; [55]. Working memory is assessed using the Y-maze [56], spatial memory using the Morris water and Barnes mazes [57], and recognition memory by using the novel object recognition test [58]. J147 has broad cognitive enhancing effects, including enhanced working memory, object recognition and spatial memory in healthy adult rodents (Fig. 4). J147 also prevented the spatial learning and memory deficits observed in AD transgenic mice (Fig. 5A and B). While there are many cognitive enhancing drugs that regulate aspects of synaptic transmission [59], J147 is a novel candidate, for it is neither a phosphodiesterase nor an acetylcholinesterase inhibitor.

There is an extensive literature showing that the BDNF signaling pathway is a major component of learning and memory [60]. BDNF is highly expressed in the hippocampus [61], and cognitive enhancement paradigms such as exercise enhance pro-BDNF, BDNF, and BDNF mRNA in the hippocampus [62], [63]. Recently, it has been argued that individuals who get regular exercise are less prone to AD [64]. Conditional BDNF gene deletion in adult mice causes impaired learning and memory in multiple assays, confirming its role in cognition [65], [66]. In addition, BDNF may enhance synaptic efficacy by promoting synaptic spine growth and stability, including the growth of new spines that increase synaptic density along neurites [67]. J147 maintains the expression of synaptophysin, a synaptic vesicle protein that is reduced in both aging and AD, and is considered an excellent marker for synapse loss (Fig. 8F) [68], [45]. J147 also prevents the loss of drebrin and synapsin-1 in the huAPP/PS1 animals (Fig. 8D and E). Drebrin is an actin binding protein that is enriched in synaptic spines that also declines with age and AD [69] and synapsin-1 is a membrane protein that is involved in synaptic vessel release [70]. These data show that J147 not only has the ability to enhance memory in both normal and AD mice, but to increase the expression of the protein substrates for memory.

Both oxidative stress and inflammatory markers are enhanced in human AD brain [71] as well as in AD transgenic mice [72]. The interpretation of biochemical markers for oxidative stress in the AD brain is, however, somewhat complicated because there are two populations of cells at the extremes of viability, one expressing the genes associated with a survival response and another reflecting activated cell death pathways. In the case of inflammation, a modest activity may be neuroprotective, for example amyloid phagocytosis by activated microglia or macrophages, while a more robust inflammatory response will be destructive. Therefore, from a therapeutic point of view, it is best to return the expression of the proteins involved to the pre-AD control level.

We examined four indicators of oxidative stress and inflammation in control, huAPP/PS1 and J147-treated huAPP/PS1 mice. Heme oxygenase 1 (HO-1) is elevated in AD [73], and in the AD transgenic animals (Fig. 7A); J147 reduces expression to below control levels. Iba-1, a marker for microglia is significantly elevated in AD mice and reduced by J147, while a toxic microglial enzyme, iNOS, is not quite significantly reduced by J147 (Fig. 7B, C). Finally, 5 LOX is a potent reactive oxygen species (ROS) producing and pro-inflammatory enzyme that is increased in AD [74], [75]. This enzyme is also increased in the transgenic mice but levels are dramatically reduced by J147 (Fig. 7D). Together these data show that J147 is able to normalize several aspects of the pro-inflammatory and pro-oxidant conditions in the transgenic animals.

Finally, AD can be viewed as a protein misfolding disease that leads to the aggregation of many proteins, including Aβ, resulting in toxicity and, ultimately, cell death. Under conditions of stress, heat shock proteins 70 and 90 are induced to enhance protein clearance. HOP-1 (also called stress-inducible protein 1) is a co-chaperone that interacts with HSP70 and HSP90 families. HOP-1 plays crucial roles in the productive folding of substrate proteins by controlling the chaperone activities of HSP70 and HSP90. Figure 8A, B, and C show that all three proteins are elevated in huAPP/PS1 mice and that J147 reduces their expression. The ability of J147 to lower the expression of heat shock proteins in AD transgenic mice again suggests that this unique compound has the ability to normalize multiple aspects of AD pathology.

### Conclusions

The above data demonstrate the potential of a new drug discovery paradigm for AD that is based upon the requirement that drug candidates be highly effective in multiple, distinct cell culture models of neurodegeneration. This approach yielded a very potent, orally active drug candidate that targets several different pathways that decline in AD. A large number of potential pharmaceutical, nutritional, and immunological therapies have been tested in clinical trials for AD, but to date they have not altered cognitive decline in humans [76]. In contrast, over 200 compounds appear to reduce plaque loads or behavioral deficits in AD transgenic mice [77] and several compounds increase the amount of BDNF in rodent brain [78]. However, none of these compounds nor any of the drugs that are in clinical trials for AD have the combination of activities of J147 in cell culture and in animals ([78]; [79]; http://clinicaltrials.gov). J147 is unique in its ability to enhance LTP, potentiate learning and memory in both normal and AD transgenic animals, and maintain synaptic proteins while at the same time reducing biochemical markers of inflammation and soluble Aβ levels in AD transgenic mice. Therefore, the range of biological activities of J147 relevant to human AD is also much more extensive than any of the compounds that have failed in clinical trials. In addition, J147 is very potent, has good medicinal chemical properties for a CNS drug [14], is apparently safe, and is orally active. Finally, unlike the current drugs approved for AD, J147 is neither an acetylcholine esterase inhibitor, an NMDA receptor antagonist, nor a phosphodiesterase inhibitor, yet it enhances cognition with a short-term treatment. Thus J147 is an exciting new compound with the potential to be an AD therapeutic by slowing disease progression through neuroprotection as well as providing immediate cognition benefits. These dual attributes improve the chances for success as a disease-modifying drug as well as in short-term AD clinical trials that use currently accepted approvable measures of outcome.

## Materials and Methods

### Materials

High glucose Dulbecco's modified Eagle's medium (DMEM) and fetal calf serum were from Invitrogen (Carlsbad, CA, USA). All other reagents were purchased from Sigma (St Louis, MO, USA). The transmembrane receptor kinase B (TrkB)-expressing HT22 cells were obtained from Dr Gerard Thiel (Homburg, Germany). The transgenic mouse line 85 was a generous gift of Dr. J.L. Jankowsky.

The primary antibodies were used at a dilution of 1∶1000 unless otherwise stated and their sources and molecular weights were as follows: Cell Signaling Technology (Danvers, MA): HOP (polyclonal, 60 kDa), Synaptophysin (polyclonal, 40 kDa) and phospho PSD95 (polyclonal, 95 kDa). Santa Cruz Biotechnology (Santa Cruz, CA): Thio Reductase-1, 1∶10000 (polyclonal, 55 kDa) and BDNF (polyclonal, 16 kDa). BD Biosciences (Rockville, MD): iNOS (monoclonal, 135 kDa) and synapsin-, 1, 1∶10000 (monoclonal, 80 kDa). Cayman Chemicals (Ann Arbor, Michigan): 5-LOX (polyclonal, 78 kDa) and 12/15 LOX (polyclonal, 75 kDa). StressMarq Biosciences Inc. (Victoria, BC, Canada): HS90 (monoclonal, 90 kDa) and HS70 (monoclonal, 70 kDa). Wako (Richmond, VA): Iba-1 (polyclonal, 16 kDa). Abcam (Cambridge, MA): Drebrin (monoclonal, 2 major bands at 50 and 100 kDa). Novus Biologicals (Littleton, CO): Homer-1 (polyclonal, 40 kDa). Sigma (St Louis, MO): anti-actin 1∶40000 (polyclonal,45 kDa). Stressgen (now Enzo Life Sciences) (Plymouth Meeting, PA): Heme oxygenase-1(HO-1) (polyclonal, 28 kDa).

### Methods

#### Ethics Statement

Experiments involving rodents were conducted following protocols approved by the Salk Institutional Animal Care and Use Committee (Protocol Number 09-025).

### Cell Culture Assays

#### Trophic Factor Withdrawal

Primary cortical neurons were prepared from 18-day-old rat embryos according to published procedures [80] and cultured at a low cell density of 1×10^6^/35 mm dish in DMEM/F12 (2 mL) containing N2 supplements (Invitrogen) and the compounds to be assayed. Viability was assayed 2 days later using a fluorescent live-dead assay (Molecular Probes, Eugene, OR, USA) and the data is presented as the percentage of input cells surviving.

#### Serum Starvation Assay

HT22 cells with or without TrkB, the brain-derived neurotrophic factor (BDNF) receptor [15], were washed three times in serum-free medium, incubated in serum-free DMEM with or without the indicated compounds for 2 days and cell viability was determined by the 3-(4,5-dimethylthiazol-2-yl)-2,5-diphenyltetrazolium bromide (MTT) assay and compared with that of cells grown in 1% serum, which inhibited cell division but maintained viability. For the MTT assay, 10 µL of 2.5 mg/mL MTT solution was added and incubated at 37°C for 4 hr and then 100 µL of solubilization solution [50% dimethylformamide and 20% sodium dodecyl sulfate (SDS), pH 4.8] was added. The next day, the absorption values at 570 nm were measured.

#### Oxytosis Assay

HT22 cells were plated at 2×10^3^ cells per well in 96-well tissue culture dishes in DMEM plus 10% fetal calf serum. The following day the test compounds were added in triplicate at appropriate concentrations. Thirty minutes after compound addition, 5 mM glutamate was added to initiate the cell death cascade. Twenty hr later, the MTT assay was performed. The results are presented as the percentage of the controls with vehicle alone.

#### In Vitro Ischemia Assay

HT22 cells were seeded onto 96-well microtiter plates at a density of 5×10^3^ cells per well in DMEM plus 10% FCS. The next day, the medium was replaced with DMEM supplemented with 7.5% dialyzed FCS and the cells were treated with 20 µM iodoacetic acid (IAA) alone. After 2 hr the medium in each well was aspirated and replaced with fresh medium without IAA but containing the test compound. 20 hr later, viability was measured by the MTT assay.

#### Glucose Starvation Assay

PC12 cells were washed three times in serum-free, glucose-free medium and exposed to glucose-free medium plus or minus the indicated compounds and cell viability determined 48 hr later using the MTT assay. The data are presented as viable cell number relative to serum-free, glucose-containing medium.

#### Aβ Toxicity to Hippocampal Neurons

Primary cultures of embryonic day 17 rat hippocampal neurons were used to assay a compound's ability to directly inhibit Aβ toxicity. The hippocampi were removed, the cells dissociated with trypsin and the cells were plated at 2×10^6^ per 35 mm polyornithine-coated tissue culture dish in 10% fetal calf serum, and 2 days later 10 µg/mL Ara C was added to inhibit glial proliferation. At day 7, 10 µM pre-aggregated Aβ_1–42_ was added with or without the test compound and viability determined 2 days later by the lactate dehydrogenase release assay [80].

#### LTP Experiments

Slice preparation and field potential recording were made as described [81]. Briefly, hippocampal slices (400 µm) were prepared from male Wistar rats (5–7 weeks old) and maintained in a chamber at 30°C, where they were continuously perfused with artificial cerebrospinal fluid consisting of (in mM); 124 NaCl, 4 KCl, 2.4 CaCl_2_, 1.3 MgSO_4_, 1.24 NaH_2_PO_4_, 26 NaHCO_3_, and 10 glucose, bubbled with 95% O_2_/5% CO_2_. The Schaffer collaterals were stimulated by a bipolar tungsten electrode positioned in the stratum radiatum of the CA1 region near the CA2/CA1 border, and the field excitatory postsynaptic potentials (fEPSPs) were recorded from the stratum radiatum of the CA1 region. The stimulus intensity was adjusted in the range of 25–55 µA to evoke fEPSPs of 50% of the maximum amplitude. Tetanic stimulation was applied at the same intensity with the test stimulation. The rising slope of fEPSP was measured as an index of synaptic efficacy. To compare the data among the groups, the averages of the fEPSP slopes 30–60 min after tetanic stimulation were calculated as an index of LTP magnitude. Data were analyzed by one-way ANOVA followed by the Dunnett's test.

### Enzyme Assays

Enzyme assays for PDIs and acetylcholine esterase were done by MDS Pharma Services (Taiwan) and Caliper Life Sciences (Hanover, MD), respectively.

### Behavioral Assays

All animal studies were carried out in strict accordance with the recommendations in the Guide for the Care and Use of Laboratory Animals of the National Institutes of Health. The protocol was approved by the Committee on the Ethics of Animal Experiments of the Salk Institute for Biological Studies and The Scripps Research Institute.

#### Rat Novel Object Recognition Test

Male Sprague-Dawley rats (175–200 grams) (Jackson Laboratory, Bar Harbor, ME) were used, and the testing was done by Behavioral Pharma, Inc. (La Jolla, CA). All rats were acclimated to the colony room for at least 2 weeks before testing, and they were tested at an average age of 8 weeks. Rats were randomly assigned across treatment groups with 12 in each group. For each dose tested, a solution of J147 was prepared in 95% ethanol, and then it was diluted with 4 volumes of polyethylene glycol 660 hydroxystearate (Solut HS15 from BASF, Florham Park, NJ) and 5 volumes of PBS. The vehicle contained the identical ratios of ethanol, Solut HS15, and PBS. All were administered orally 60 min before the training trial at a volume of 10 ml/kg of body weight. Galantamine was dissolved in 10% DMSO, and it was administered i.p. at 3 mg/kg 20 min before training. The test was performed as described by Bourtchouladze et al. [82]. Briefly, on day 1, rats were habituated to a circular open field arena for 1 hr in cage groups of four. Twenty-four hr later, individual rats were placed back in the same arena, which now contained two identical objects for a 15-min training trial. On day 3, vehicle-, J147, or galantamine-treated rats were individually placed back in the same arena in the presence of both the familiar object (i.e., previously explored) and a novel object. The spatial positions of the objects were counter balanced between subjects. Each animal's test trial was recorded, and the first 10 min of each session were scored. Object recognition was computed by using the following formula: Time spent exploring the novel object divided by total time spent exploring both objects ×100. Data were analyzed by a one-way ANOVA followed by post hoc comparisons with Fisher's test.

#### Mouse Behavioral Testing

For the Barnes maze, Y maze and NOR tests, male C57BL/6J mice were provided J147 in standard rodent chow (N = 12) or were given control feed (N = 12) starting at 6 weeks of age. Body weights were determined before and during the experimental sequence. Barnes maze testing was initiated 2 weeks into J147 exposure. Y maze and NOL testing took place 7 and 8 wks into exposure, respectively.

#### Barnes Maze

The Barnes maze apparatus is an opaque Plexiglas disc 75 cm in diameter elevated 58 cm above the floor by a tripod. Twenty holes, 5 cm in diameter, are located 5 cm from the perimeter, and a black Plexiglas escape box (19×8×7 cm) is placed under one of the holes. Distinct spatial cues are located all around the maze and are kept constant throughout the study.

On the first day of testing, a training session was performed, which consists of placing the mouse in the escape box for one minute. After the one minute habituation period, the first session was started. At the beginning of each session, the mouse was placed in the middle of the maze in a 10 cm. high cylindrical black start chamber. After 10 seconds the start chamber was removed, a buzzer (80 dB) and a light (400 lux) were turned on, and the mouse was set free to explore the maze. The session ended when the mouse entered the escape tunnel or after 3 min elapsed. When the mouse entered the escape tunnel, the buzzer was turned off and the mouse was allowed to remain in the dark for one minute. When the mouse did not enter the tunnel by itself it was gently put in the escape box for one minute. The tunnel was always located beneath the same hole (stable within the spatial environment), which was randomly determined for each mouse. Mice were tested once a day for 9 days for the acquisition portion of the study.

Six weeks later the mice were tested again with the escape box in the original position (retention test). This allowed for the examination of long-term memory. Finally, on the day after this test, the escape tunnel was moved to a new location (90 degrees from the original position) and the behavior of the mouse was recorded. This is called the reversal test and it allowed for the examination of the strategies adopted by the mouse to locate the new tunnel location.

Each session was videotaped and scored by an experimenter blind to the treatment group of the mouse. Measures recorded included latencies to escape, the number of errors made per session and the strategy employed by the mouse to locate the escape tunnel. Errors are defined as nose pokes and head deflections over any hole that did not have the tunnel beneath it. Search strategies are determined by examining each mouse's daily session and classifying it into one of three categories: 1) Random search strategy – localized hole searches separated by crossings through the center of the maze, 2) Serial search strategy – systematic hole searches (every hole or every other hole) in a clockwise or counterclockwise direction, or 3) Spatial search strategy - reaching the escape tunnel with both error and distance (number of holes between the first hole visited and the escape tunnel) scores of less than or equal to 3. Barnes maze data were analyzed by repeated measures analysis of variance and group differences in individual time blocks and the retention and reversal trials were compared using Student's t-tests. Two proportion z-tests were used to compare strategies employed between groups.

#### Novel Object Location Test

Mice were tested in an open field apparatus (79×79×50.8 cm) with a small bottle placed in one location for four 5 min trials. For the 5^th^ trial, the object was moved to a different spatial location. The inter-trial interval was 1 min. All behavior was video recorded and then scored for number of object contacts (touching with nose or nose pointing at object and within 0.5 cm of object). The data are presented as recognition index (see above) and analyzed using the Student's t-test.

#### Y-Maze Test

For the Y maze test for spontaneous alternations the same mouse groups were used as with the Barnes maze on NOL tests. Spontaneous alternation, the tendency to alternate free choices in a Y-maze (3 arms), is a model for studying working memory in mice. A single 5 min test was performed in which each mouse was placed in the center of the Y. Arm entries were recorded by video camera and the total number of arm entries and the order of entries were determined. Spontaneous alternations are defined as consecutive triplets of different arm choices. Y maze data were analyzed using Student's t-tests.

### huAPPswe/PS1ΔE9 Transgenic Mice

#### Animals

The APP/PS1 transgenic mice (line 85) have been previously characterized [83]. The line 85 mice carry two transgenes, the mouse/human chimeric APP/Swe, linked to Swedish FAD and human PS1ΔE9. At three months of age male transgenic mice and their wild type littermates were fed high fat diet (Harlan Teklad, Madison, WI) with and without J147 (200 ppm). Mouse body weights and food consumption were measured weekly, and there were no significant differences between the groups.

#### Morris Water Maze

Spatial memory was determined in 9 month old huAPPswe/PS1 transgenic mice fed J147 at 200 ppm in food for the previous 5 months using the Morris water maze (MWM). The MWM procedure was 4 trials per day for 5 consecutive days. For each trial, mice were placed in the pool at 1 of 4 start locations. The starting locations were separated by 90° and will be identified as south, west, north, and east. Mice started a trial once from each of the 4 possible start locations on each day. The goal platform was positioned 45 cm from the outside wall in the south quadrant of the maze for all groups. The latency to find and mount the hidden platform was used as the primary dependent variable. Swimming speeds were recorded to assess drug-induced motor effects. Mice were given a maximum of 120 s to find the hidden platform. If the mice failed to find the platform after 120 s, they were placed on the platform by the experimenter. All mice remained on the platform for 15 s before being placed in a heated incubator (30°C) between trials. Personnel evaluating animals in the MWM were blinded to the injury and drug treatments of each animal. A mean daily latency to find the goal platform during MWM testing was computed for each mouse. On day 6, a probe test was done in which the platform was removed and the time spent in the platform quadrant determined. Analysis of latency times was done by ANOVA of repeated measures. Days are within the subjects factor and group is between the subjects factor. A significance level of *P*<0.05 was considered significant.

#### Tissue Preparation and Immunoblotting

Hippocampal tissue samples were homogenized in 10 volumes of RIPA lysis buffer (50 mM Tris, pH 7.5, 150 mM NaCl, 0.1% sodium dodecyl sulfate and 0.5% deoxycholate, and 1% NP40) containing a cocktail of protease and phosphatase inhibitors [20 mg/ml each of pepstatin A, aprotinin, phosphoramidon, and leupeptin; 0.5 mM 4-(2-aminoethyl) benzenesulfonyl fluoride hydrochloride; 1 mM EGTA; 5 mM fenvalerate; and 5 mM cantharidin]. Samples were sonicated (2×10 s) and centrifuged at 100,000×g for 60 min at 4°C.

Protein concentrations in the cell extracts were determined using the BCA protein assay (Pierce). Equal amounts of protein were solubilized in 2.5× SDS-sample buffer, separated on 12% or 10–20% SDS-polyacrylamide gels, transferred to Immodulin P and immunoblotted with the antibodies indicated in Materials. For western blots, protein levels were normalized to actin levels. ANOVA analysis with the Tukey post hoc test was used to determine differences between means for Western blot analysis.

#### Immunohistochemistry

Brains were fixed with 4% paraformaldehyde in 100 mM sodium tetraborate, pH 9.5, for 3 h, cryoprotected with 20% sucrose-potassium-PBS (KPBS), and cryostat sectioned into coronal (30 µm) sections. Sections were submerged in 0.3% H_2_O_2_ for 10 min to eliminate endogenous peroxidase activity and treated with 1% borate to eliminate free paraformaldehyde. Sections were incubated with primary antibody in 0.3% Triton X-100 in KPBS plus 2% filtered serum or BSA overnight at 4°C, and with primary antibodies (1∶1000) in 0.3% Triton X-100 for 1 hr at room temperature. After incubation with secondary antibody and ABC reagent (Vector Laboratories), sections were developed using metal-enhanced DAB solution. Sections were mounted to slides, dried, dehydrolyzed, treated with xylene, and covered using dibutyl phthalate xylene. Images were captured by a Zeiss digital camera connected to a Zeiss VivaTome microscope, and image analysis on sections was performed using Axiovision software.

Quantification of amyloid plaque burden was based on the image captured by immunohistochemical staining with antibody 6E10. Sections of each mouse cortex and hippocampus were imaged together and the areas and densities of the plaques in the hippocampus only were measured by the Image J software (NIH). The total counts of Aβ plaques in sections per six mouse brains of each group were determined in an unbiased fashion.

#### Aβ ELISA

Aβ 1–40 and 1–42 levels in hippocampal lysate were analyzed using the Aβ_1–40_ and Aβ_1–42_ ELISA kits from Invitrogen (# KHB3481 and # KHB3442, respectively). All kit reagents were allowed to reach room temperature before use. Standards were prepared according to manufacturers guidelines and samples were diluted as follows; RIPA fractions were diluted 1∶10 for both Aβ_1–40_ and Aβ_1–42_; and RIPA insoluble fractions were diluted 1∶2000 for Aβ_1–40_ and 1∶5000 for Aβ_1–42_. 50 µl of Aβ peptide standards and samples were added in duplicate to 96 well plates pre-coated with mAb to the NH_2_ terminus region of Aβ. Plates were incubated at 4°C overnight and then 50 µl of Hu Aβ40 or Aβ42 detection antibody was added to each well except the chromogen blanks. Plates were incubated at room temperature with gentle shaking for 3 hours and then washed 4 times with provided wash buffer. At this time, 100 µl of Anti-Rabbit IgG HRP working solution was added to each well except the chromogen blanks for 30 min at room temperature. Wells were then washed as before four times and incubated with 100 µl of Stabilized chromogen for 25 min at room temperature in the dark. Stop solution was then added at 100 µl to each well followed by reading the absorbance of each well at 450 nm. Curve fitting software was used to generate the standard curve where a four parameter algorithm provided the best standard curve fit. The concentrations of the samples were calculated from the standard curve and multiplied by the dilution factor.

### Chemistry

#### Diversity Synthesis

m-Anisaldehyde (50 mmol, 1 eq.) was dissolved in dichloromethane (300 ml) under nitrogen and 2,4 dimethylphenyl hydrazine hydrochloride (50 mmol, 1 eq.) was added and mixed for 30 min. Trifluoroacetic acid (TFA) (100 mmol, 2 eq.) was added and stirred for an additional hr. Finally, triacetoxyborohydride (60 mmol, 1.2 eq.) was added along with 200 ml of additional dichloromethane, and the reaction stirred overnight under nitrogen. Five hundred ml of water was added to the orange reaction mixture, and stirred for 1 hr to dissolve the borohydride salts. The layers were separated in a funnel, and the organic layer washed three times with 0.1 M sodium bicarbonate, separated, and dried. The products were separated into eight spots on silica then layer plates, (hexane-ethyl acetate solvent), and the most active fraction in the oxytosis, Aβ toxicity and TFW assays run on HPLC (C18 reverse phase 20 to 100% acetonitrile linear gradient in 0.1% TFA) and assayed.

### 1-(2,4-dimethylphenyl)-2-(3-methoxybenzylidene)hydrazine hydrochloride (3)

**Figure 10:**
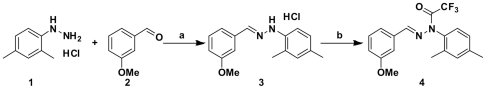
Preparation of J147. J147 was prepared as described in the chemistry section of the materials and methods. The reagents and conditions for each step are as follows: (a) EtOH, rt, 1 h, 90%; (b) (CF_3_CO)_2_O, Et_3_N, 30 min, 10°C, 77%.

The preparation of J147 is shown in Figure 10. A mixture of **1**(115 g, 0.66 mol, 1.0 eq.) and **2**(90 g, 0.66 mol, 1.0 eq.) in EtOH (500 mL) was stirred at RT under N_2_ for 1 h. A light brown solid precipitated and was filtered. The solid was washed with EtOH and methyl butyl ether, dried in vacuo to afford **3** as a light brown solid (151 g) with a yield of 90%. This unstable compound was used directly in the next step.

### (E)-N-(2,4-dimethylphenyl)-2,2,2-trifluoro-N′-(3-methoxybenzylidene) acetohydrazide (4)

To a solution of **3**(70 g, 0.24 mol, 1.0 eq.), Et_3_N(85 mL, 0.61 mol, 2.5 eq.) in anhydrous Ch_2_Cl_2_(400 mL) trifluoroacetic anhydride (34 mL, 0.24 mol, 1.0 eq.) was added drop-wise under N_2_ while cooled with ice-water. After stirring for 30 min under the same conditions, the mixture was concentrated in a rotary evaporator, and filtered through a short silica gel column using petroleum-EtOAc(1∶1) as the eluent solvent. The filtrate was concentrated and re-crystallized from methyl butyl ether to afford **4** as a white solid (65 g) at a yield of 77%.

^1^H NMR (300 MHz, CDC13): δ/ppm = 7.32∼6.98(m, 8H), 3.84(s, 3H), 2.43(s, 3H), 2.10(s, 3H). MS-ESI: cal. 250; found: 251(M+H^+^), 273(M+Na^+^), 405(M+Na^+^+MeOH).

## References

[1] Pangalos MN, Schechter LE, Hurko O (2007). Drug development for CNS disorders: strategies for balancing risk and reducing attrition.. Nat Rev Drug Discov.

[2] Herrup K (2010). Reimagining Alzheimer's disease–an age-based hypothesis.. J Neurosci.

[3] Frautschy SA, Cole GM (2010). Why pleiotropic interventions are needed for Alzheimer's disease.. Mol Neurobiol.

[4] Ganesan A (2008). The impact of natural products upon modern drug discovery.. Curr Opin Chem Biol.

[5] Cole GM, Teter B, Frautschy SA (2007). Neuroprotective effects of curcumin.. Adv Exp Med Biol.

[6] Lim GP, Chu T, Yang F, Beech W, Frautschy SA (2001). The curry spice curcumin reduces oxidative damage and amyloid pathology in an Alzheimer transgenic mouse.. J Neurosci.

[7] Schubert DR, Liu Y (2002). Method for preventing and/or decreasing amyloid production with polycyclic compounds..

[8] Liu Y, Dargusch R, Maher P, Schubert D (2008). A broadly neuroprotective derivative of curcumin.. J Neurochem 105.

[9] Haigis MC, Yankner BA (2010). The aging stress response.. Mol Cell.

[10] Shia CS, Tsai SY, Kuo SC, Hou YC, Chao PD (2009). Metabolism and pharmacokinetics of 3,3′,4′,7-tetrahydroxyflavone (fisetin), 5-hydroxyflavone, and 7-hydroxyflavone and antihemolysis effects of fisetin and its serum metabolites.. J Agric Food Chem.

[11] Abe K, Takayanagi M, Saito H (1990). Effects of recombinant human basic FGF and its modified protein CS23 on survival of primary cultured neurons from various regions of fetal rat brain.. Japan J Pharmacol.

[12] Tan S, Schubert D, Maher P (2001). Oxytosis: A novel form of programmed cell death.. Curr Topics Med Chem.

[13] Yankner BA, Duffy LK, Kirschner DA (1990). Neurotrophic and neurotoxic effects of amyloid beta protein: reversal by tachykinin neuropeptides.. Science.

[14] Pajouhesh H, Lenz GR (2005). Medicinal chemical properties of successful central nervous system drugs.. NeuroRx.

[15] Rossler OG, Giehl KM, Thiel G (2004). Neuroprotection of immortalized hippocampal neurones by brain-derived neurotrophic factor and Raf-1 protein kinase: role of extracellular signal-regulated protein kinase and phosphatidylinositol 3-kinase.. J Neurochem.

[16] Maher P, Parker L, Sies H, Eggersdorfer M, Cardenas E (2010). Modulation of multiple pathways involved in the maintenance of neuronal function by fisetin.. Micronutrients and Brain Health.

[17] Winkler BS, Sauer MW, Starnes CA (2003). Modulation of the Pasteur effect in retinal cells: implications for understanding compensatory metabolic mechanisms.. Exp Eye Res.

[18] Maher P, Salgado KF, Zivin JA, Lapchak PA (2007). A novel approach to screening for new neuroprotective compounds for the treatment of stroke.. Brain Res.

[19] Chen J, Zacharek A, Zhang C, Jiang H, Li Y (2005). Endothelial nitric oxide synthase regulates brain-derived neurotrophic factor expression and neurogenesis after stroke in mice.. J Neurosci.

[20] Olin D, MacMurray J, Comings DE (2005). Risk of late-onset Alzheimer's disease associated with BDNF C270T polymorphism.. Neurosci Lett.

[21] Tapia-Arancibia L, Aliaga E, Silhol M, Arancibia S (2008). New insights into brain BDNF function in normal aging and Alzheimer disease.. Brain Res Rev.

[22] Pezet S, Malcangio M (2004). Brain-derived neurotrophic factor as a drug target for CNS disorders.. Expert Opin Ther Targets.

[23] Aisen PS, Andrieu S, Sampaio C, Carrillo M, Khachaturian ZS (2011). Report of the task force on designing clinical trials in early (predementia) AD.. Neurology.

[24] Ennaceur A, Delacour J (1988). A new one-trial test for neurobiological studies of memory in rats. 1: Behavioral data.. Behav Brain Res.

[25] Zhang HT, Huang Y, Suvarna NU, Deng C, Crissman AM (2005). Effects of the novel PDE4 inhibitors MEM1018 and MEM1091 on memory in the radial-arm maze and inhibitory avoidance tests in rats.. Psychopharmacology (Berl).

[26] Woodruff-Pak DS, Vogel RW, Wenk GL (2001). Galantamine: effect on nicotinic receptor binding, acetylcholinesterase inhibition, and learning.. Proc Natl Acad Sci U S A.

[27] Sarnyai Z, Sibille EL, Pavlides C, Fenster RJ, McEwen BS (2000). Impaired hippocampal-dependent learning and functional abnormalities in the hippocampus in mice lacking serotonin(1A) receptors.. Proc Natl Acad Sci U S A.

[28] Young JW, Powell SB, Risbrough V, Marston HM, Geyer MA (2009). Using the MATRICS to guide development of a preclinical cognitive test battery for research in schizophrenia.. Pharmacol Ther.

[29] Bach ME, Hawkins RD, Osman M, Kandel ER, Mayford M (1995). Impairment of spatial but not contextual memory in CaMKII mutant mice with a selective loss of hippocampal LTP in the range of the theta frequency.. Cell.

[30] Jankowsky JL, Melnikova T, Fadale DJ, Xu GM, Slunt HH (2005). Environmental enrichment mitigates cognitive deficits in a mouse model of Alzheimer's disease.. J Neurosci.

[31] Savonenko A, Xu GM, Melnikova T, Morton JL, Gonzales V (2005). Episodic-like memory deficits in the APPswe/PS1dE9 mouse model of Alzheimer's disease: relationships to beta-amyloid deposition and neurotransmitter abnormalities.. Neurobiol Dis.

[32] van Groen T, Kiliaan AJ, Kadish I (2006). Deposition of mouse amyloid beta in human APP/PS1 double and single AD model transgenic mice.. Neurobiol Dis.

[33] Lalonde R, Kim HD, Maxwell JA, Fukuchi K (2005). Exploratory activity and spatial learning in 12-month-old APP(695)SWE/co+PS1/DeltaE9 mice with amyloid plaques.. Neurosci Lett.

[34] Vorhees CV, Williams MT (2006). Morris water maze: procedures for assessing spatial and related forms of learning and memory.. Nat Protoc.

[35] Wang F, Chen H, Sun X (2009). Age-related spatial cognitive impairment is correlated with a decrease in ChAT in the cerebral cortex, hippocampus and forebrain of SAMP8 mice.. Neurosci Lett.

[36] Harrison FE, Hosseini AH, McDonald MP, May JM (2009). Vitamin C reduces spatial learning deficits in middle-aged and very old APP/PSEN1 transgenic and wild-type mice.. Pharmacol Biochem Behav.

[37] Krafft GA, Klein WL (2010). ADDLs and the signaling web that leads to Alzheimer's disease.. Neuropharmacology.

[38] Bishop NA, Lu T, Yankner BA (2010). Neural mechanisms of ageing and cognitive decline.. Nature.

[39] Cameron B, Landreth GE (2010). Inflammation, microglia, and Alzheimer's disease.. Neurobiol Dis.

[40] Dore S (2002). Decreased activity of the antioxidant heme oxygenase enzyme: implications in ischemia and in Alzheimer's disease.. Free Radic Biol Med.

[41] Schipper HM, Song W, Zukor H, Hascalovici JR, Zeligman D (2009). Heme oxygenase-1 and neurodegeneration: expanding frontiers of engagement.. J Neurochem.

[42] Radmark O, Werz O, Steinhilber D, Samuelsson B (2007). 5-Lipoxygenase: regulation of expression and enzyme activity.. Trends Biochem Sci.

[43] Phillis JW, Horrocks LA, Farooqui AA (2006). Cyclooxygenases, lipoxygenases, and epoxygenases in CNS: their role and involvement in neurological disorders.. Brain Res Rev.

[44] Di Domenico F, Sultana R, Tiu GF, Scheff NN, Perluigi M (2010). Protein levels of heat shock proteins 27, 32, 60, 70, 90 and thioredoxin-1 in amnestic mild cognitive impairment: an investigation on the role of cellular stress response in the progression of Alzheimer disease.. Brain Res.

[45] Reddy PH, Mani G, Park BS, Jacques J, Murdoch G (2005). Differential loss of synaptic proteins in Alzheimer's disease: implications for synaptic dysfunction.. J Alzheimers Dis.

[46] Zhao L, Ma QL, Calon F, Harris-White ME, Yang F (2006). Role of p21-activated kinase pathway defects in the cognitive deficits of Alzheimer disease.. Nat Neurosci.

[47] Dawson GR, Seabrook GR, Zheng H, Smith DW, Graham S (1999). Age-related cognitive deficits, impaired long-term potentiation and reduction in synaptic marker density in mice lacking the beta-amyloid precursor protein.. Neuroscience.

[48] Cunha C, Brambilla R, Thomas KL (2010). A simple role for BDNF in learning and memory?. Front Mol Neurosci.

[49] Sato M, Suzuki K, Nakanishi S (2001). NMDA receptor stimulation and brain-derived neurotrophic factor upregulate homer 1a mRNA via the mitogen-activated protein kinase cascade in cultured cerebellar granule cells.. J Neurosci.

[50] Yoshimura Y, Shinkawa T, Taoka M, Kobayashi K, Isobe T (2002). Identification of protein substrates of Ca(2+)/calmodulin-dependent protein kinase II in the postsynaptic density by protein sequencing and mass spectrometry.. Biochem Biophys Res Commun.

[51] Guerreiro RJ, Gustafson DR, Hardy J (2010). The genetic architecture of Alzheimer's disease: beyond APP, PSENs and APOE.. Neurobiol Aging.

[52] Figurov A, Pozzo-Miller LD, Olafsson P, Wang T, Lu B (1996). Regulation of synaptic responses to high-frequency stimulation and LTP by neurotrophins in the hippocampus.. Nature.

[53] Allen GL, Kirasic KC, Rashotte MA, Haun DB (2004). Aging and path integration skill: kinesthetic and vestibular contributions to wayfinding.. Percept Psychophys.

[54] Bach ME, Barad M, Son H, Zhuo M, Lu YF (1999). Age-related defects in spatial memory are correlated with defects in the late phase of hippocampal long-term potentiation in vitro and are attenuated by drugs that enhance the cAMP signaling pathway.. Proc Natl Acad Sci U S A.

[55] Barnes CA (1979). Memory deficits associated with senescence: a neurophysiological and behavioral study in the rat.. J Comp Physiol Psychol.

[56] Aggleton JP, Hunt PR, Rawlins JN (1986). The effects of hippocampal lesions upon spatial and non-spatial tests of working memory.. Behav Brain Res.

[57] Morris RG, Garrud P, Rawlins JN, O'Keefe J (1982). Place navigation impaired in rats with hippocampal lesions.. Nature.

[58] Broadbent NJ, Gaskin S, Squire LR, Clark RE (2010). Object recognition memory and the rodent hippocampus.. Learn Mem.

[59] Lanni C, Lenzken SC, Pascale A, Del Vecchio I, Racchi M (2008). Cognition enhancers between treating and doping the mind.. Pharmacol Res.

[60] Minichiello L (2009). TrkB signalling pathways in LTP and learning.. Nat Rev Neurosci.

[61] Dugich-Djordjevic MM, Peterson C, Isono F, Ohsawa F, Widmer HR (1995). Immunohistochemical visualization of brain-derived neurotrophic factor in the rat brain.. Eur J Neurosci.

[62] Berchtold NC, Castello N, Cotman CW (2010). Exercise and time-dependent benefits to learning and memory.. Neuroscience.

[63] Griesbach GS, Hovda DA, Gomez-Pinilla F (2009). Exercise-induced improvement in cognitive performance after traumatic brain injury in rats is dependent on BDNF activation.. Brain Res.

[64] Radak Z, Hart N, Sarga L, Koltai E, Atalay M (2010). Exercise plays a preventive role against Alzheimer's disease.. J Alzheimers Dis.

[65] Monteggia LM, Barrot M, Powell CM, Berton O, Galanis V (2004). Essential role of brain-derived neurotrophic factor in adult hippocampal function.. Proc Natl Acad Sci U S A.

[66] Gorski JA, Balogh SA, Wehner JM, Jones KR (2003). Learning deficits in forebrain-restricted brain-derived neurotrophic factor mutant mice.. Neuroscience.

[67] Engert F, Bonhoeffer T (1999). Dendritic spine changes associated with hippocampal long-term synaptic plasticity.. Nature.

[68] Calhoun ME, Jucker M, Martin LJ, Thinakaran G, Price DL (1996). Comparative evaluation of synaptophysin-based methods for quantification of synapses.. J Neurocytol.

[69] Dun XP, Chilton JK (2010). Control of cell shape and plasticity during development and disease by the actin-binding protein Drebrin.. Histol Histopathol.

[70] Yamagata Y (2003). New aspects of neurotransmitter release and exocytosis: dynamic and differential regulation of synapsin I phosphorylation by acute neuronal excitation in vivo.. J Pharmacol Sci.

[71] Bonda DJ, Wang X, Perry G, Nunomura A, Tabaton M (2010). Oxidative stress in Alzheimer disease: A possibility for prevention.. Neuropharmacology.

[72] Dumont M, Wille E, Stack C, Calingasan NY, Beal MF (2009). Reduction of oxidative stress, amyloid deposition, and memory deficit by manganese superoxide dismutase overexpression in a transgenic mouse model of Alzheimer's disease.. FASEB J.

[73] Orozco-Ibarra M, Chirino YI, Pedraza-Chaverri J (2006). Role of hemeoxygenase-1 in the neurodegenerative disorders.. Rev Neurol.

[74] Yao Y, Clark CM, Trojanowski JQ, Lee VM, Pratico D (2005). Elevation of 12/15 lipoxygenase products in AD and mild cognitive impairment.. Ann Neurol.

[75] Ikonomovic MD, Abrahamson EE, Uz T, Manev H, Dekosky ST (2008). Increased 5-lipoxygenase immunoreactivity in the hippocampus of patients with Alzheimer's disease.. J Histochem Cytochem.

[76] Wollen KA (2010). Alzheimer's disease: the pros and cons of pharmaceutical, nutritional, botanical, and stimulatory therapies, with a discussion of treatment strategies from the perspective of patients and practitioners.. Altern Med Rev.

[77] Zahs KR, Ashe KH (2010). ‘Too much good news’ - are Alzheimer mouse models trying to tell us how to prevent, not cure, Alzheimer's disease?. Trends Neurosci.

[78] Coma M, Sereno L, Da Rocha-Souto B, Scotton TC, Espana J (2010). Triflusal reduces dense-core plaque load, associated axonal alterations and inflammatory changes, and rescues cognition in a transgenic mouse model of Alzheimer's disease.. Neurobiol Dis.

[79] Golde TE, Schneider LS, Koo EH (2011). Anti-abeta therapeutics in Alzheimer's disease: the need for a paradigm shift.. Neuron.

[80] Behl C, Davis JB, Lesley R, Schubert D (1994). Hydrogen peroxide mediates amyloid beta protein toxicity.. Cell.

[81] Abe K, Kimura H (1996). The possible role of hydrogen sulfide as an endogenous neuromodulator.. J Neurosci.

[82] Bourtchouladze R, Lidge R, Catapano R, Stanley J, Gossweiler S (2003). A mouse model of Rubinstein-Taybi syndrome: Defective long-term memory is ameliorated by inhibitors of phosphodiesterase 4.. Proc Natl Acad Sci USA.

[83] Jankowsky JL, Younkin LH, Gonzales V, Fadale DJ, Slunt HH (2007). Rodent A beta modulates the solubility and distribution of amyloid deposits in transgenic mice.. J Biol Chem.

[84] Lapchak PA, Schubert DR, Maher PA (2011). Delayed treatment with a novel neurotrophic compound reduces behavioral deficits in rabbit ichemic stroke.. J Neurochem.

